# Heterogeneous Slowdown of Dynamics in the Condensate of an Intrinsically Disordered Protein

**DOI:** 10.1021/acs.jpclett.4c02142

**Published:** 2024-11-01

**Authors:** Saumyak Mukherjee, Lars V. Schäfer

**Affiliations:** Center for Theoretical Chemistry, Ruhr University Bochum, 44780 Bochum, Germany

## Abstract

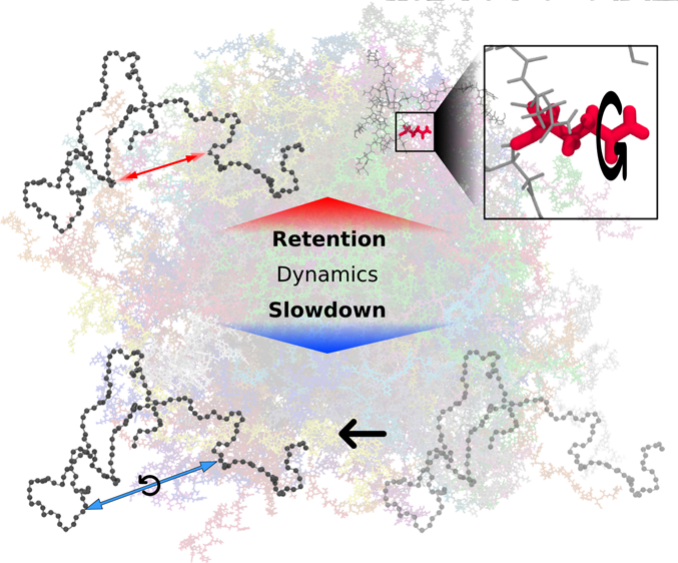

The high concentration of proteins and other biological macromolecules inside biomolecular condensates leads to dense and confined environments, which can affect the dynamic ensembles and the time scales of the conformational transitions. Here, we use atomistic molecular dynamics (MD) simulations of the intrinsically disordered low complexity domain (LCD) of the human fused in sarcoma (FUS) RNA-binding protein to study how self-crowding inside a condensate affects the dynamic motions of the protein. We found a heterogeneous retardation of the protein dynamics in the condensate with respect to the dilute phase, with large-amplitude motions being strongly slowed by up to 2 orders of magnitude, whereas small-scale motions, such as local backbone fluctuations and side-chain rotations, are less affected. The results support the notion of a liquid-like character of the condensates and show that different protein motions respond differently to the environment.

The discovery of membraneless organelles (MLOs) has changed cellular biophysics,^1^ challenging the traditional view that cellular compartmentalization is exclusively mediated by lipid-bound organelles and vesicles. MLOs are high-density liquid droplets formed through the process of liquid–liquid phase separation (LLPS)^2−6^ of proteins, particularly intrinsically disordered proteins (IDPs),^7,8^ and RNA.^9^ They play critical roles in orchestrating various cellular processes.^3,10−13^ For example, the biochemical reactions occurring in MLO compartments are crucial for transcription, stress response, synaptic activity, RNA splicing, receptor-mediated signaling, and mitosis.^14−22^

Due to their ability to concentrate biomolecules, MLOs are described as biomolecular condensates or biocondensates.^23^ The concentrations of proteins and/or nucleic acids inside the condensates are typically higher than 100 mg mL^–1^.^24−27^ This leads to increased viscosity inside the droplets, which can be 3–6 orders of magnitude higher than in dilute solutions.^28^ Consequently, the diffusion of biomolecules in condensates is strongly retarded.^24,25,29−33^ For example, an up to 500-fold decrease of translational diffusion was reported for condensates of the intrinsically disordered low complexity domain (LCD) of the human fused in sarcoma (FUS) RNA-binding protein.^25^

To fulfill their biological functions, biocondensates need to ensure a high local concentration of certain biomolecules. At the same time, these biomolecules need to retain their dynamic nature as much as possible, and such liquid-like character is also crucial for the exchange of molecules between condensates and their surrounding. The dynamic nature of condensates partly originates from the high degree of conformational flexibility of the constituent IDPs, also fostered by the relatively high water content of the droplets.^25,34,35^ Driven by their plasticity and the lack of a membrane, the condensates can rapidly form, reshape, and adapt their properties and, hence, efficiently interact and exchange molecules with their surroundings.^36^ The exact link between the dynamic nature of condensates and their biological functions can sometimes be difficult to determine though, and it may strongly depend upon the particular system under investigation.^13,37^ In a striking recent example, Fischer et al. showed that transcription factor condensates with more liquid-like character lead to higher gene expression levels than stiffer condensates.^38^

The dynamic and broad conformational ensembles of IDPs, as dictated by their amino acid sequences, lead to dynamic multivalent interactions that play a key role in driving LLPS and stabilizing biocondensates.^5,39^ In general, the flat free-energy landscapes of IDPs imply the lack of a stable persistent conformation but a highly dynamic conformational ensemble that is characterized by rapid transitions between a multitude of metastable minima.^40,41^ This plasticity of IDPs^42^ fosters dynamic interactions in the condensates, which include mechanisms such as folding upon binding, the formation of highly dynamic biomolecular complexes, conformational selection, and fly casting.^43−46^ Some of these mechanisms involve the transient formation of (local) structure, e.g., via the restriction of certain dynamic modes.^28^ The increased viscosity in highly concentrated condensates is expected to further restrict the conformational dynamics of the proteins, although the droplets retain a liquid-like character and the proteins of which they are composed can still exhibit rapid chain dynamics.^47−49^

Zheng et al. used molecular dynamics (MD) simulations to investigate the multivalent interactions that govern LLPS of FUS-LCD.^47^ They showed that ion partitioning between dense and dilute phases is driven by the charge distribution along the protein chain.^47^ Furthermore, the MD simulations of Zheng et al. also showed that, despite the high concentration inside the droplets, the protein molecules remain mobile and that the dynamic self-association of the proteins is driven by a combination of non-specific interactions as well as hydrogen bonds, salt bridges, and π–π and cation−π interactions.^47^

In a combined experimental/computational study, Galvanetto et al.^48^ used fluorescence spectroscopy and MD simulations to investigate the prothymosin-α/histone H1 (ProTα/H1) system. ProTα has ultrahigh affinity toward H1 due to strong electrostatic interactions of the two highly charged proteins (the structural charges of ProTα and H1 are −44 and +53, respectively, which strongly differ from the small net charge of FUS-LCD of −2). A main finding of that study was that nanosecond time-scale dynamics are retained in the condensate despite a ca. 300-fold higher viscosity than in the dilute phase, a conclusion that agrees with the results reported by Zheng et al. for FUS-LCD. Interestingly, a recent nuclear magnetic resonance (NMR) relaxation study of the measles virus *N*_TAIL_ protein, an IDP that undergoes LLPS, showed that, while sampling of the protein backbones is not strongly affected in the dense phase, librational, backbone torsional, and segmental (or chain-like) dynamics are considerably slower,^49^ a finding that needs to be reconciled with the results of Galvanetto et al. Along the same lines, a time-resolved fluorescence spectroscopy study found that α-synuclein has reduced chain flexibility under LLPS conditions compared to dilute solution.^50^ However, no specific dynamic protein modes were identified, and the effects on backbone and side-chain motions were not separately addressed. All in all, we conclude that further studies are needed to characterize and understand the dynamic behavior of biomolecules in condensates in more detail.

Here, we approach biomolecular condensates from a fundamental biophysical chemistry perspective and investigate the effect of the dense environment of a biomolecular condensate on the protein dynamics. Single-component condensates formed by the LCD of human FUS are used as a model system to investigate which dynamic modes of the protein are affected to what extent in the dense condensate environment compared to dilute conditions. FUS is physiologically important for RNA shearing and transport, DNA repair, micro-RNA processing, and gene transcription and regulation.^51−53^ The LCD region is primarily responsible for driving LLPS.^24,25,27,54,55^ It encompasses the N-terminal 163 residues of the protein and is rich in glutamine and serine residues, tyrosines, and also glycines, which are known to be important for condensate formation via LLPS.^56^

All-atom MD simulations were used to study the dynamics of the protein backbone and side-chains of FUS-LCD in condensates and compare them to dilute conditions (snapshots of the systems are shown in Figure S1 of the Supporting Information). To understand the condensate effects on the dynamics of the protein chains, both the backbone and side-chain motions were investigated. First, the overall translational dynamics of the FUS-LCD chains were characterized by calculating the mean-squared displacements (MSDs) of the protein atoms. From a linear fit to the MSD of the center of mass of the FUS-LCD chains, we obtained a rough estimate of the translational diffusion coefficient of 0.26 × 10^–3^ nm^2^/ns (Figure S2 of the Supporting Information), which is within the range of experimental values reported by Fawzi and co-workers (from 0.17 to 0.4 × 10^–3^ nm^2^/ns).^24,25^

Figure 1A shows the MSD, averaged over all individual backbone C_α_ atoms in the FUS-LCD chains, plotted as a function of the lag time. In the condensate, an average translational slowdown relative to the dilute phase of up to 2 orders of magnitude is found (the black dashed line in Figure 1A represents the mean MSD of the C_α_ atoms in the condensate multiplied by 100). This result is also in line with previous simulations showing strongly retarded overall diffusion of proteins in crowded environments.^57−60^ To analyze this slowdown in more detail, MSDs of groups of increasing numbers of consecutive C_α_ atoms in the FUS-LCD chain were calculated, with group sizes ranging from a single C_α_ atom to the whole chain (163 C_α_ atoms).
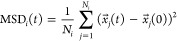
1In eq 1,  and  denote the position vectors of the *j*th C_α_ atom at time origin and at a lag time *t* later, respectively. Each group of size *N*_*i*_ (the number of C_α_ atoms in the *i*th group) has 163 – *N*_*i*_ + 1 instances or subgroups, and the MSD was calculated by averaging over all subgroups.

**Figure 1:**
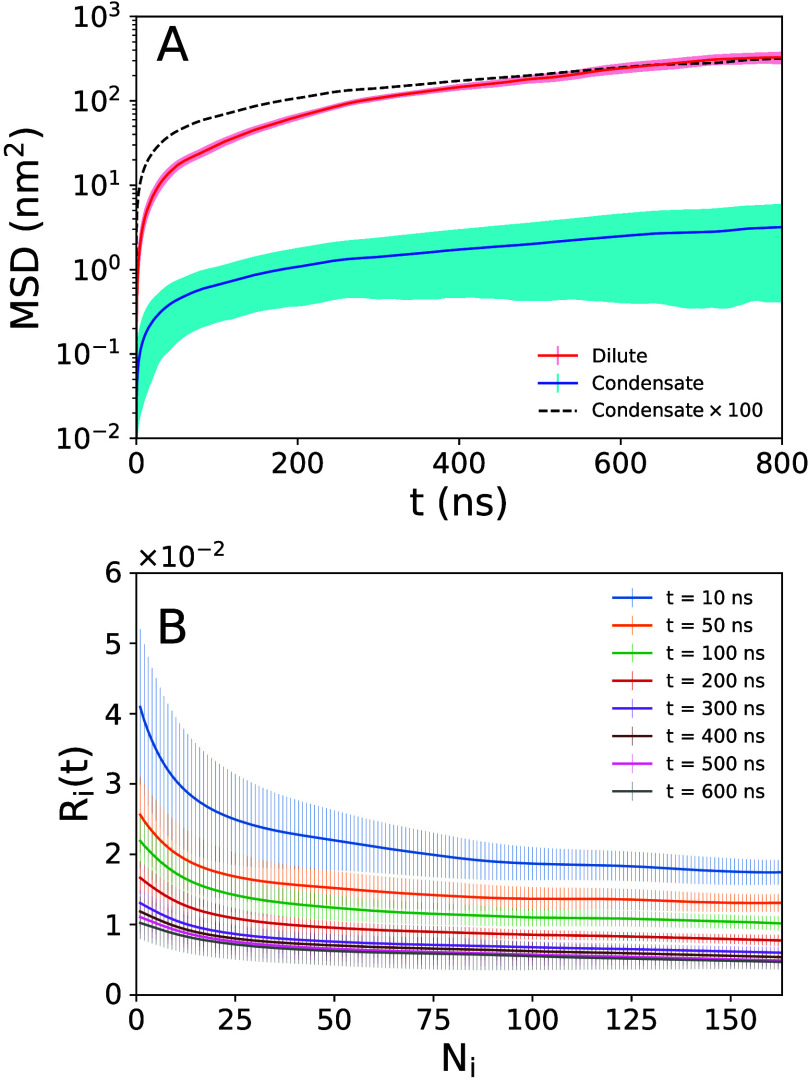
(A) MSDs of C_α_ atoms in the protein backbones in the dilute (red) and condensate (blue) systems plotted as a function of the lag time *t*. The solid lines represent the mean MSD, and the error bars denote the standard deviations over all of the C_α_ atoms considered. The black dashed line represents the condensate MSD multiplied by 100. (B) Ratio of the mean MSDs of C_α_ atoms in the condensate and dilute systems at selected lag times (10, 50, 100, 200, 300, 400, 500, and 600 ns) plotted against the number of C_α_ atoms in a group. The error bars denote the standard deviations over the eight protein chains in the simulation system.

Figure 1B shows the ratio of MSDs in the condensate and dilute systems as a function of *N*_*i*_ at selected lag times
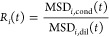
2A small value of *R*_*i*_(*t*) indicates a strong slowdown of dynamics in the condensate phase. As shown in Figure 1B, the dynamical retardation is more pronounced at longer lag times compared to shorter lag times. Additionally, the slowdown is stronger for larger groups of atoms, whereas smaller groups of atoms at shorter lag times experience the least dynamical retardation. The differences in *R*_*i*_(*t*) diminish for longer lag times, indicating that the retardation as a function of lag time is nonlinear and that, beyond a certain point, the dynamics are similarly slowed down.

Our findings show that motions of larger groups of atoms on larger length scales (as expected at longer times) through the viscous condensate environment are subject to stronger retardation. This is in line with the theoretical expectation that the friction experienced by a (spherical) probe particle upon moving through a macromolecular environment is reduced due to an entropic depletion effect,^61^ leading to an effective viscosity that depends upon the size of the diffusing molecule relative to the mesh spacing of the surrounding macromolecular network.^33,62^

The above analysis provides a measure of the retardation of the translational motions of the protein backbone in the condensate. To more closely understand this dynamic slowdown, the reorientation motions of the protein backbone were analyzed separately from the side-chains. To this end, the reorientation of the vectors between two C_α_ atoms at varying sequence separations was studied. First, the orientational autocorrelation functions (ACFs)
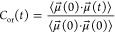
3were calculated for the C_α_–C_α_ vectors μ⃗(*t*) at different separations ( = 1–162), averaged over all possible instances of . The brackets ⟨...⟩ indicate the average over all time step differences *t*. The ACF in eq 3 describes the reorientation motion of the C_α_–C_α_ vectors via the angle θ_*t*_ formed by a vector during the time interval *t*. The mean ACFs were fitted to biexponential functions (eq 4), and the mean orientational time scales (eq 5) were obtained by integration
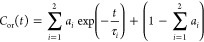
4
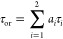
5The amplitudes *a*_*i*_ were normalized to obtain relaxations between 1 and 0. ACFs for selected C_α_–C_α_ separations are shown in Figure S3 of the Supporting Information. The applicability of the biexponential fit was decided by calculating the coefficient of determination (*R*^2^) values at each separation and comparing mono-, bi-, and triexponential fits (Figure S4 of the Supporting Information). The triexponential fits were not superior to the biexponential fits; therefore, the biexponentials were used.

The same approach was taken to quantify the magnitude of distance fluctuations of the C_α_–C_α_ vectors
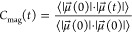
6where  and  are the magnitudes of the vectors at time origin and at a lag time *t*, respectively. Also, in this case, biexponential functions accurately described the relaxations (Figures S5 and S6 of the Supporting Information).

Panels A and B of Figure 2 show the orientational time scales for the dilute (τ_dil_^or^) and condensate (τ_cond_^or^) systems, respectively. The ratio of the two time constants is shown in Figure 2C. Panels D–F of Figure 2 show the analogous graphs for the C_α_–C_α_ distance fluctuations. In this case, the correlation times are denoted by τ_dil_^mag^ and τ_cond_^mag^ for the dilute and condensate phases, respectively.

**Figure 2:**
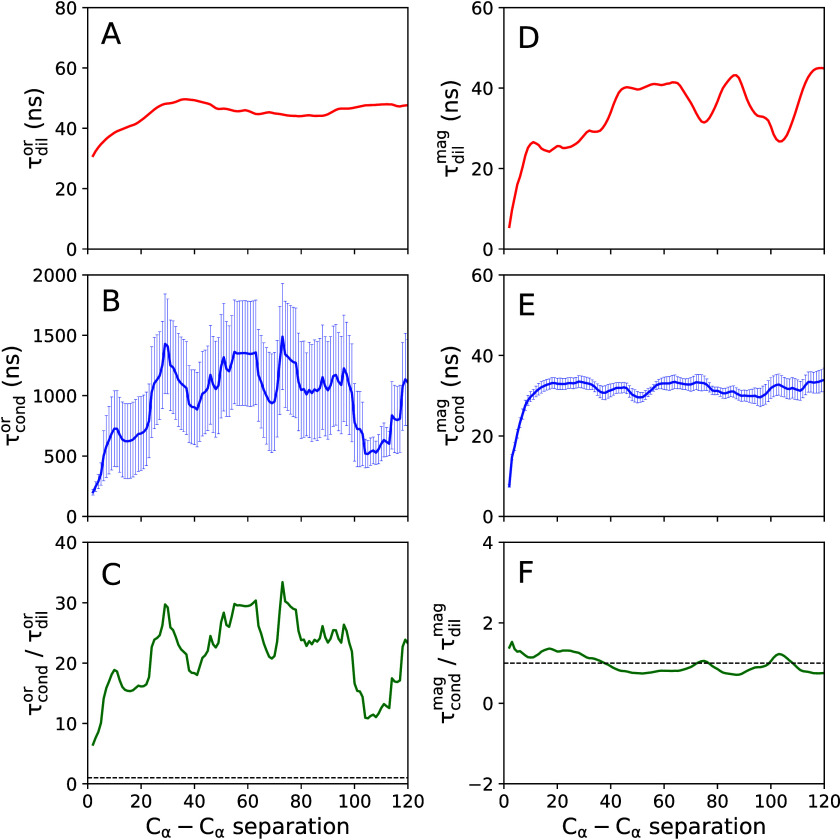
Protein backbone dynamics for FUS-LCD chains. (A and B) Time scales of orientational dynamics as a function of C_α_–C_α_ separation for the dilute and condensate systems, respectively. (C) Ratio of these two time scales. (D–F) Analogous properties for the C_α_–C_α_ distance fluctuations. The error bars denote the standard errors over the eight proteins in the condensate system.

Figure 2A shows that retardation of orientational backbone dynamics is observed with an increase in . For larger length scales (sequence separation ), the value of τ_dil_^or^ plateaus. A qualitatively similar behavior is also observed for τ_dil_^mag^ (Figure 2D). A smooth  dependence is found for τ_cond_^mag^ (Figure 2E), while τ_cond_^or^ (Figure 2B) shows larger variations.

The time-scale ratio depicted in Figure 2C illustrates that the reorientation motions of the backbone are slowed down by factors of approximately 5–15 (about 1 order of magnitude) in the condensate compared to the dilute system. In contrast, the C_α_–C_α_ distance dynamics exhibit no noticeable slowdown in the condensate system (Figure 2F). This apparent contradiction can be resolved by considering the nature of dynamical variables analyzed. While the reorientation dynamics of the protein backbone involves motions of backbone atoms through a dense and viscous environment, the distance magnitude fluctuations pertain to the local motions of the C_α_ atoms around their average positions. Thus, in the context of the above results on the retardation of global protein translational dynamics, we conclude that protein dynamics involving large-scale motions are more strongly affected by the condensate environment.

Next, the motions of the amino acid side-chains were investigated in terms of rotamer jumps. The side-chain rotamer dynamics were analyzed by computing the normalized time ACFs *C*_χ_(*t*) of the dihedral angles χ_*i*_ (*i* = 1, 2, and 3, depending upon the length of the side-chain).
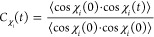
7For each amino acid occurring in the FUS-LCD sequence that at least has the χ_1_ dihedral (Asn, Asp, Gln, Ser, Thr, and Tyr), for each χ_*i*_, the ACFs (eq 7) were averaged over all of the side-chains present in the system. These mean ACFs were fitted by biexponential functions (eq 4) and integrated (eq 5) to obtain the correlation times, which are plotted in Figure 3. The individual ACFs are shown in Figures S7 and S8 of the Supporting Information. Biexponential functions accurately describe the ACFs (Figure S9 of the Supporting Information).

**Figure 3:**
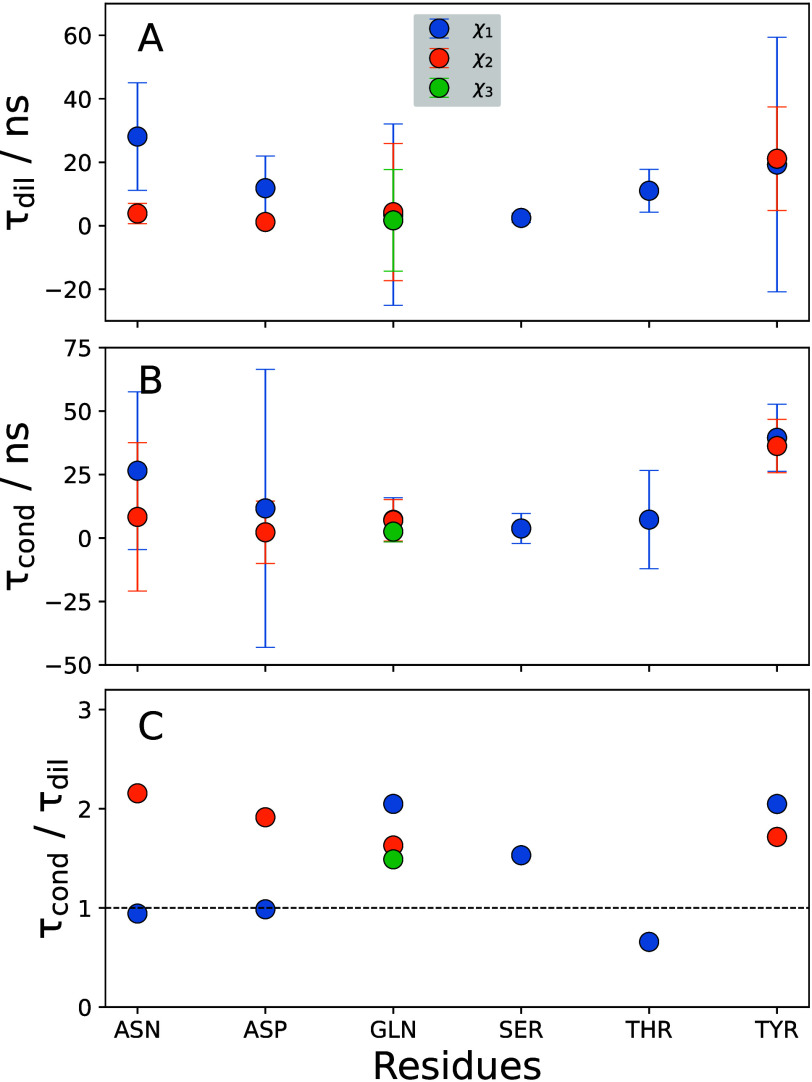
Time scales of side-chain dihedral angle rotations in the (A) dilute and (B) condensate systems. (C) Ratio of the time constants in the two systems. The χ_1_, χ_2_, and χ_3_ dihedral angles of the respective residues are plotted in blue, orange, and green, respectively. The error bars denote the standard errors over all of the respective residues present in the system.

Panels A and B of Figure 3 present the time scales in the dilute (τ_dil_) and condensate (τ_cond_) systems, respectively. The ratio of τ_cond_ and τ_dil_ is shown in Figure 3C. The side-chain dynamics are not significantly slowed in the condensate. At first sight, it may seem surprising that rotamer jumps are not affected even for the tyrosine side-chains, which are known to play a distinct role for mediating residue–residue interactions in FUS condensates.^47^ Averaged over all side-chains and dihedral angles (χ_1_, χ_2_, and χ_3_), the retardation is about 2-fold. This small change is similar to the backbone C_α_–C_α_ magnitude fluctuations, supporting the notion that the time scales of the small-amplitude motions are not affected much in the condensate. As described above, at short length scales, the entropic depletion effect reduces the effective viscosity of the macromolecular network, and hence, fast local motions do not experience a strong retardation.

Finally, we aimed at obtaining a more complete picture and analyzed how the heterogeneous retardation of the protein dynamics in the condensate described above influences the dynamics of the surrounding water molecules. As protein and water motions are coupled^63−70^ and because water motions on a local scale are fast (typically in the picoseconds regime), we expected them to couple most strongly to the fast local small-amplitude protein motions and, thus, to find a similar modest condensate-related slowdown as was found for the local protein motions, i.e., about 2-fold. To test that hypothesis, we probed the reorientation motions (rotations) of the water molecules in the protein hydration layer (PHL), which was defined as the region within 0.5 nm of the protein surface. To quantify the time scales of water rotations, we computed the time ACFs of the dipole moment vector of each water molecule, tracking only those water molecules that remained within the PHL for the duration of the analysis. In this case, μ⃗(*t*) in eq 3 denotes the normalized dipole moment vector of a single water molecule [for a rigid water model, as used in this work, an O–H bond vector (and not the dipole moment) can be used for the analysis]. As described above, the ACFs were fitted to biexponential functions and integrated to obtain the rotational correlation times. The distributions obtained for the dilute and condensate phases are plotted in Figure 4A.

**Figure 4:**
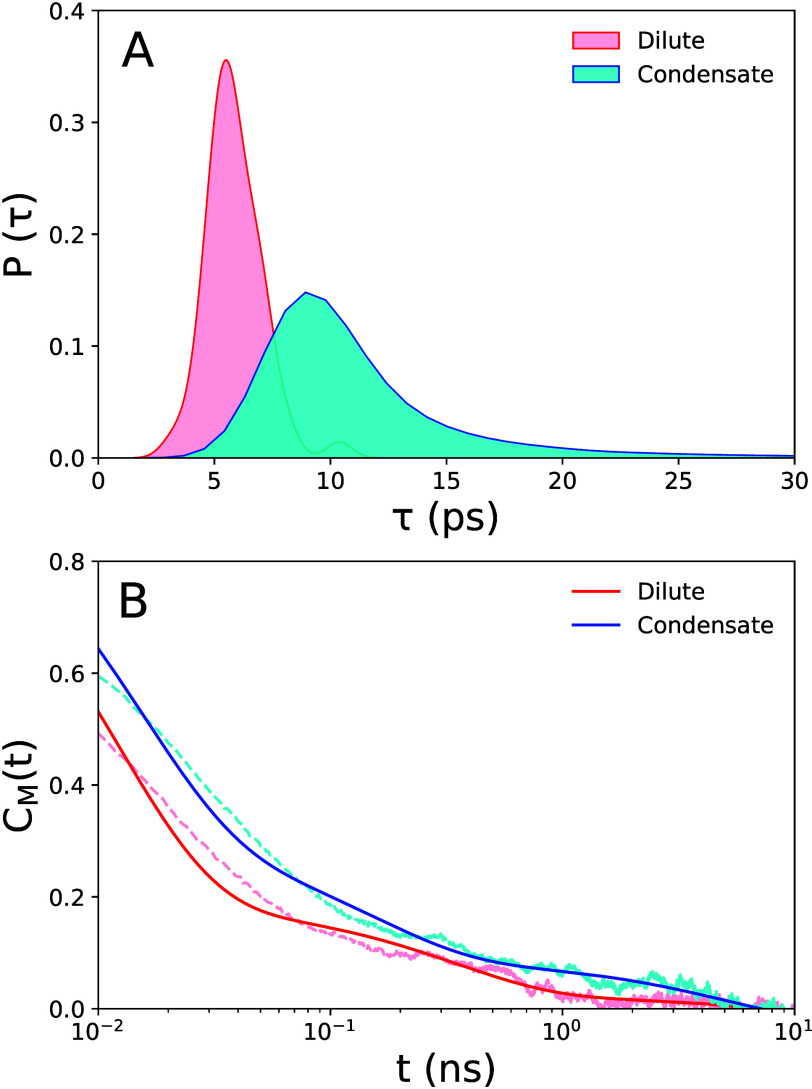
Dynamics of the PHL in the dilute (red) and condensate (blue) phases. The PHL is defined by a cutoff distance of 0.5 nm from the protein. (A) Distributions of rotational correlation times. (B) ACFs of total dipole moment (and triexponential fits, solid lines) of PHL water molecules in the dilute and condensate phases plotted on a semi-log scale.

Figure 4A shows that the time-scale distribution for the condensate system is broader and slightly shifted in comparison to the dilute phase, indicating a partial slowdown in the rotational dynamics of water in the PHL in the condensate. The mean correlation times for the dilute and condensate systems are 6 and 12 ps, respectively. Hence, the water reorientations in the condensate are slowed down by a factor of 2.

While the above analysis provides a measure of the dynamical slowdown at the level of single water molecules, a more global picture can be obtained by comparing the collective relaxation of the entire protein hydration layer, as described by the ACF of the total dipole moment *C*_M_(*t*)
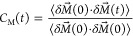
8In eq 8, the total dipole moment fluctuation is denoted by , where  is the total dipole moment vector of the PHL,  denotes the time-averaged dipole moment, and  is the dipole moment of the *i*th water molecule in the PHL. *C*_M_(*t*) of the dilute (red) and condensate (blue) phases are plotted in Figure 4B. The dashed lines represent the raw ACFs, whereas the solid lines denote their fits. In this case, a triexponential function provided better fits (*R*_dil_^2^ = 0.93 and *R*_cond_^2^ = 0.96) than a biexponential function (*R*_dil_^2^ = 0.91 and *R*_cond_^2^ = 0.92). The average correlation times thus obtained are 101 and 670 ps for the dilute and condensate systems, respectively. The hydration water analysis thus shows that single-molecule rotational water motions are slowed down 2-fold, while overall collective dynamics of the ensemble of hydration water molecules are slower and affected to a greater extent by the condensate environment. These results are in qualitative agreement with recent vibrational spectroscopy studies,^71,72^ which showed that local vibrational probes, such as the amide backbone C=O stretch or the arginine side-chain C–N stretches, are slowed modestly in the condensate environment compared to the dilute phase (about 2–6 fold, in agreement with our above results, e.g., of the local side-chain motions). The dynamic retardation of these local protein modes is transmitted to the surrounding water in the PHL via the H-bond network.

The interpretation of the relevance of the (overall only relatively modest) retardation of the water dynamics in the condensate phase in the context of biological function is challenging. However, we point out that slow water dynamics and a concomitant stiffening of the water H-bond network are generally associated with lower water entropy.^73−77^ The associated solvation-related contributions to the thermodynamic driving forces of condensate formation can be sizable in magnitude, especially when the water molecules are constrained by a strongly confined environment in the condensate.^78,79^ However, even at protein concentrations as high as 300 mg/mL and beyond, the water volume fraction in the condensate is still relatively large (above 60%), ensuring that the condensates retain a sufficient liquid-like character to maintain a high degree of biomolecular dynamics.

In conclusion, in this work, we used atomistic MD simulations to probe the dynamic slowdown of an intrinsically disordered protein and the water in its hydration layer in biocondensates, using one-component FUS-LCD condensates as a model system. The crowded milieu in the dense condensate phase slows down both protein and water dynamics but to markedly different extents depending upon the length scale of the motions involved. The retardation of dynamics is non-universal and heterogeneous, with dynamic modes that involve large-scale motions being affected more strongly than small-scale local fluctuations. The most significant dynamical retardation in the condensate of up to almost 2 orders of magnitude is found for the translational motions of the protein backbone. The slowdown increases with increasing number of atoms involved in the motion; that is, collective large-amplitude motions are affected the most. For small-amplitude motions, such as C_α_ distance fluctuations and side-chain dihedral rotations, the dynamical slowdown by the condensate is not very pronounced. However, the orientational dynamics of C_α_–C_α_ vectors, which require movement of the protein backbone chain through the condensate interior, are found to be slowed down by 1 order of magnitude.

Taken together, this work provides detailed microscopic insights into how slow translational diffusion on a global scale is linked to fast local structural dynamics. The latter might be important for the biological function of FUS, for example, by fostering molecular recognition processes, such as FUS binding to RNA or protein factors involved with transcription initiation and regulation. The connection found here between the dynamic slowdown and the time and length scales of the associated protein motions might be transferable to other biocondensates and, thus, have more general implications beyond the specific system studied in this work. The precise link between protein dynamics and biological function is very case-specific, but it has been shown for many biomolecular systems that dynamics are required for function. It is therefore essential for the proteins to retain their dynamic personalities^80^ in dense biocondensates, as exemplified in this work.

## Supporting Information Available

Methods (system setup and simulation details), MSD over lag time plot and linear fit of translational diffusion coefficient, selected ACFs for C_α_–C_α_ reorientation dynamics for selected separations in the condensate system, *R*^2^ values plotted against C_α_–C_α_ separations for reorientation dynamics in dilute and condensate systems for different fitting functions, selected ACFs for C_α_–C_α_ distance fluctuations for selected separations in the condensate system, *R*^2^ values plotted against C_α_–C_α_ separations for distance fluctuations in dilute and condensate systems for different fitting functions, ACFs of side-chain rotamer dynamics in dilute and condensate systems, and *R*^2^ values for different fitting functions in the side-chain dynamic ACFs (PDF)
Transparent Peer Review report available (PDF)
