# Supplementary material for: Heterogeneous Slowdown of Dynamics in the Condensate of an Intrinsically Disordered Protein
Source: J Phys Chem Lett. 2024 Nov 1;15(45):11244–51. doi: 10.1021/acs.jpclett.4c02142 (PMC11571228; doi:10.1021/acs.jpclett.4c02142)
Supplement: Supplementary file 1 — jz4c02142_si_001.pdf [file jz4c02142_si_001.pdf]

Supporting Information: Heterogeneous Slowdown of Dynamics in the Condensate of an Intrinsically Disordered Protein

Saumyak Mukherjee and Lars V. Schäfer*

Center for Theoretical Chemistry, Ruhr University Bochum, D-44780 Bochum, Germany

E-mail: lars.schaefer@ruhr-uni-bochum.de

Methods

System preparation

The intrinsically disordered low complexity domain (LCD) of the human Fused in Sarcoma (FUS) RNA-binding protein (UniProt ID: P3563, residues 1 to 163 of a total 526 residues) was simulated in this study. The initial atomic coordinates of FUS-LCD were generated using AlphaFold,[S1,S2] which predicted an extended conformation without any secondary structure.

As explained in detail in our previous work,[S3] the initial equilibrated condensate system at a protein concentration of 350 mg ml^{-1} was obtained from coarse-grained (CG) MD simulations with the Martini 2.2[S4] force field. Scaled protein-protein Lennard-Jones (LJ) interactions were used to maintain the liquid-like dynamical features of the condensate, as suggested by Benayad et al.[S5] Eight copies of the coarse-grained FUS-LCD chain were inserted in a cubic periodic simulation box along with 4366 CG water beads (1 Martini water bead corresponds to 4 atomistic water molecules) and 16 sodium ions to neutralize the net charge of the simulation box.

The system was energy minimized and simulated for 1 μs under NpT conditions at T = 300 K and p = 1 bar. The resulting box dimensions were 8.9 nm. Temperature and pressure were maintained using a weak coupling thermostat and barostat,[S6] respectively. The coarse-grained simulations were performed using the recommended "new rf" settings,[S7] which included the use of a 1.1 nm cut-off for the non-bonded interactions and a 20 fs time step to integrate the equations of motion.

The CG simulations enabled fast equilibration of the protein configurations. However, realistic dynamical data (time scales) can not be obtained from this approach. Hence, the CG simulations were only used for initial equilibration but not for analysis. All production simulations were performed with the all-atom force field amber99SB-disp.[S8] First, the systems obtained from CG equilibration were back-mapped to the all-atom level using the `initram.sh` and `backward.py` scripts.[S9] The resulting atomistic proteins were solvated with 16509 water molecules. 79 sodium and 63 chloride ions were added to neutralize the charges from the protein chains and to have a salt concentration of 150 mM, corresponding to physiological ion concentrations. The protein, water and ions were described by the a99SB-disp force field (and the corresponding 4-point water model derived from TIP4P-D),[S8] which was shown to be a good choice for FUS in a recent study.[S10]

A reference dilute system was also simulated. It included a single FUS-LCD chain in 45681 water molecules along with 134 sodium and 132 chloride ions in a 11.2 nm cubic periodic box. The salt concentration in this case was also 150 mM. Snapshots of the dilute and condensate systems are shown in Figs. S1A and S1B respectively.

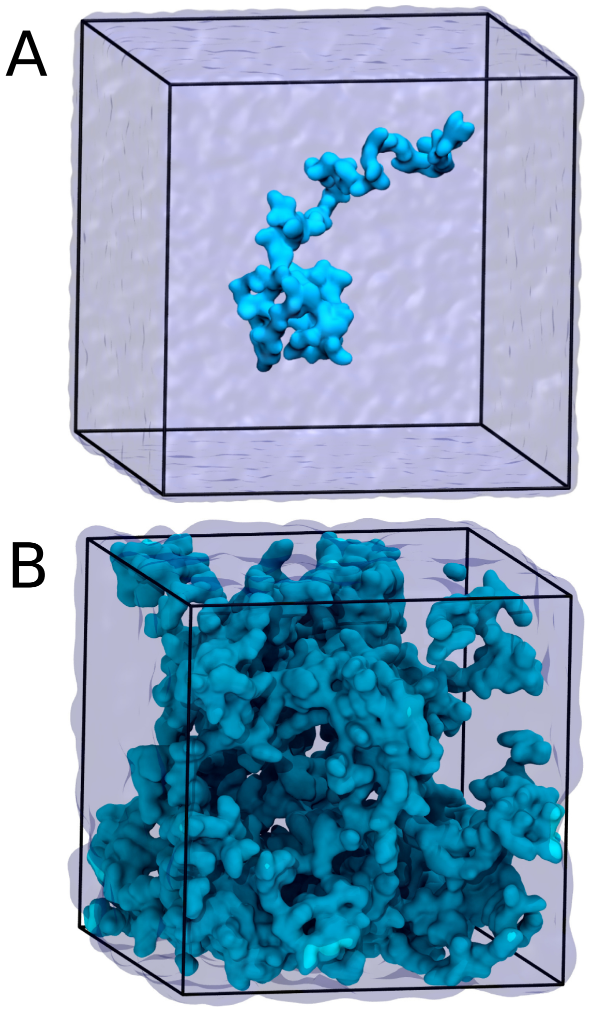

Figure S1: Snapshots of the equilibrated simulation boxes of (A) dilute and (B) condensate systems.

Simulation details

All simulations were performed using the GROMACS (version 2021.1) molecular dynamics simulation package.[S11] Both the systems were first energy minimized using the steepest descent algorithm and then equilibrated with harmonic position restraints on the protein backbone for 10 ns (restraining force constants of 1000 kJ mol^{-1} nm^{-2}) under NpT conditions at T = 300 K and p = 1 bar. This was followed by further NpT equilibration under the same conditions for 100 ns without any position restraints. Thereafter, the systems were equilibrated for 100 ns at constant volume, followed by the final production runs for 1 μs

in the NVT ensemble. Coordinates were saved to disk every 10 ps. For the analysis of water dynamics, 100 ns production runs were performed with a data saved to disk every 1 ps. In these simulations, the velocity rescaling thermostat with a stochastic term[S12] and the Berendsen barostat[S6] were used to control temperature and pressure, respectively.

All atomistic MD simulations were done using the leapfrog integrator with a time step of 2 fs. Protein bonds and internal degrees of water molecules were constrained using the LINCS and SETTLE algorithms, respectively. Short-range Coulomb and Lennard-Jones interactions were calculated up to an interparticle distance cut-off of 1.0 nm. Long-range electrostatic interactions were treated with the particle-mesh Ewald method[S13] with a grid spacing of 0.12 nm and cubic spline interpolation.

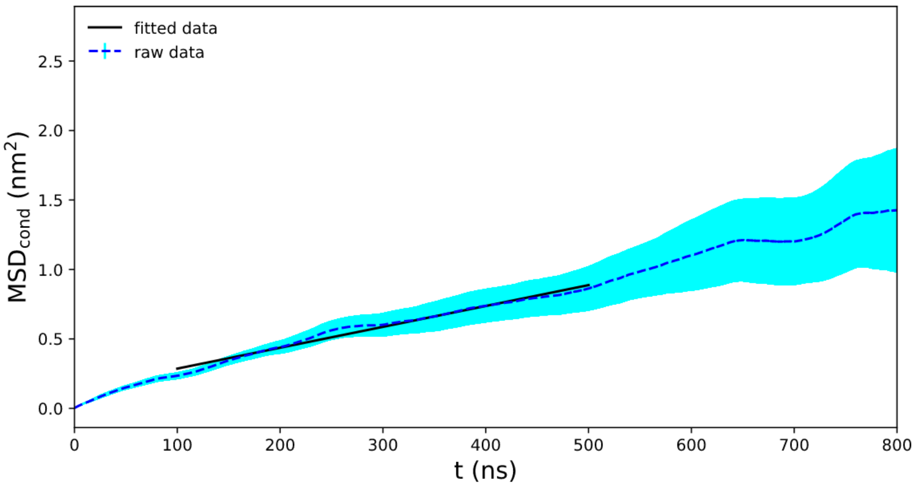

Figure S2: Mean squared displacement (MSD) of the centers of mass of the FUS-LCD chains in the condensate simulation system. The dashed line represents the raw data, averaged over the eight proteins in the simulation box. The cyan shaded area shows the standard deviation over the eight proteins. The solid line depicts the linear fit from 100 ns to 500 ns; the slope is $6D$, with D the apparent translational diffusion coefficient. The obtained value of $D = 0.26 \cdot 10^{-3}$ nm^2/ns is within the range of values from FRAP and NMR experiments reported by Fawzi and coworkers[S14,S15] (0.17 to 0.4 $\cdot 10^{-3}$ nm^2/ns). However, we consider it to be only a rather rough estimate, because the fit assumes a linear relationship between MSD and lag time and thus neglects subdiffusive behavior, which is expected to play a role for the confined motions in the condensate. Furthermore, we did not correct for finite box size effects[S16] because the viscosity of the simulation box is unknown. Such correction would increase the value slightly.

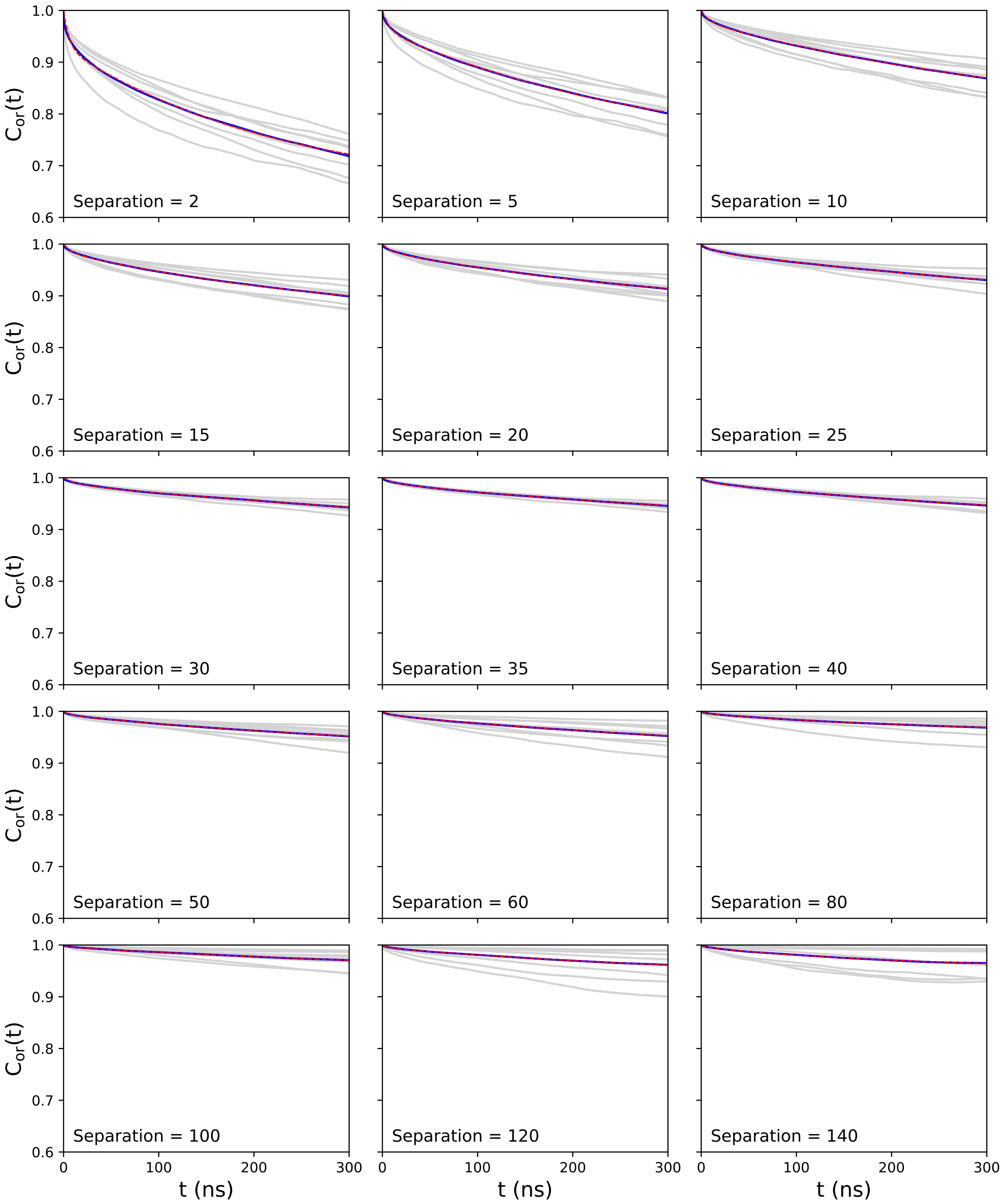

Figure S3: Orientational autocorrelation functions of the $C_\alpha - C_\alpha$ vectors at selected sequence separations in the condensate system. The light grey curves represent the 8 individual proteins in the system. The bold blue line is the average ACF over the 8 individual ones. The dashed red lines show the biexponential fits.

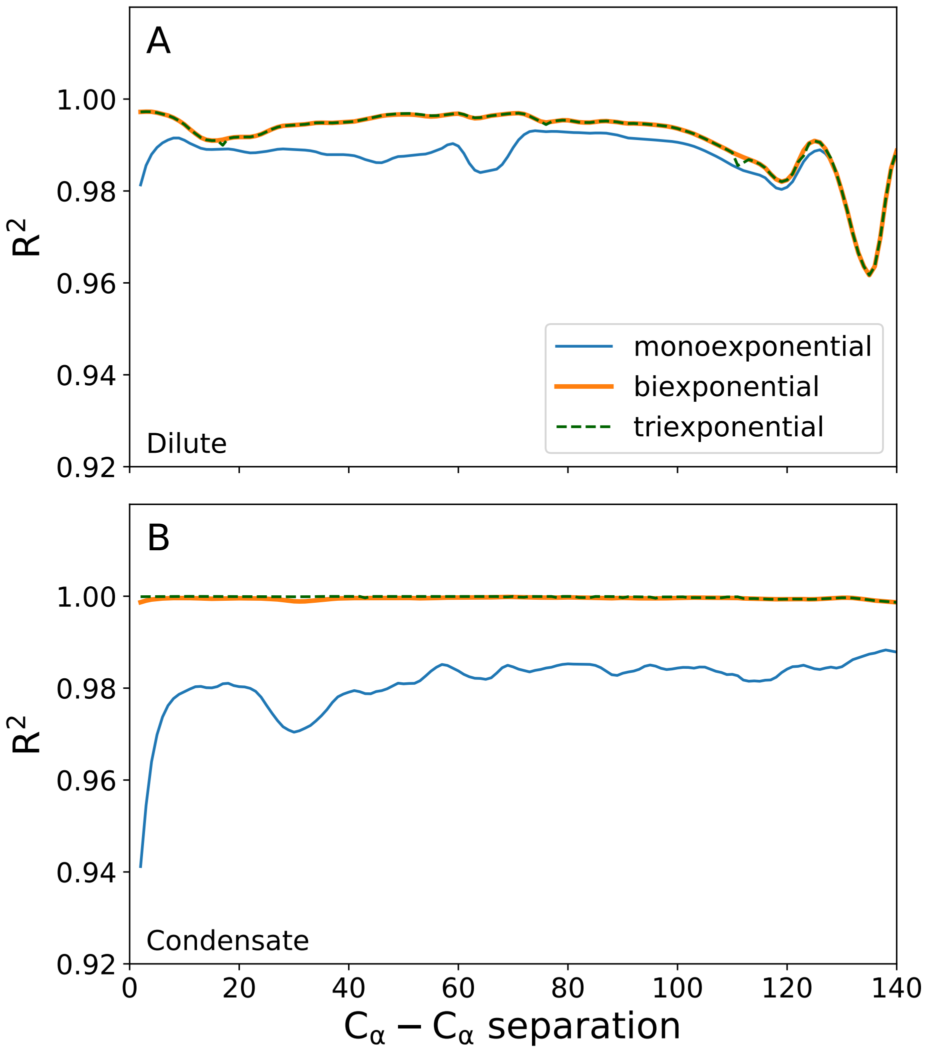

Figure S4: Coefficient of determination (R^2) for mono-, bi-, and triexponential fits of the ACFs of $C_\alpha - C_\alpha$ vector orientation dynamics as a function of $C_\alpha - C_\alpha$ separation in (A) dilute and (B) condensate systems.

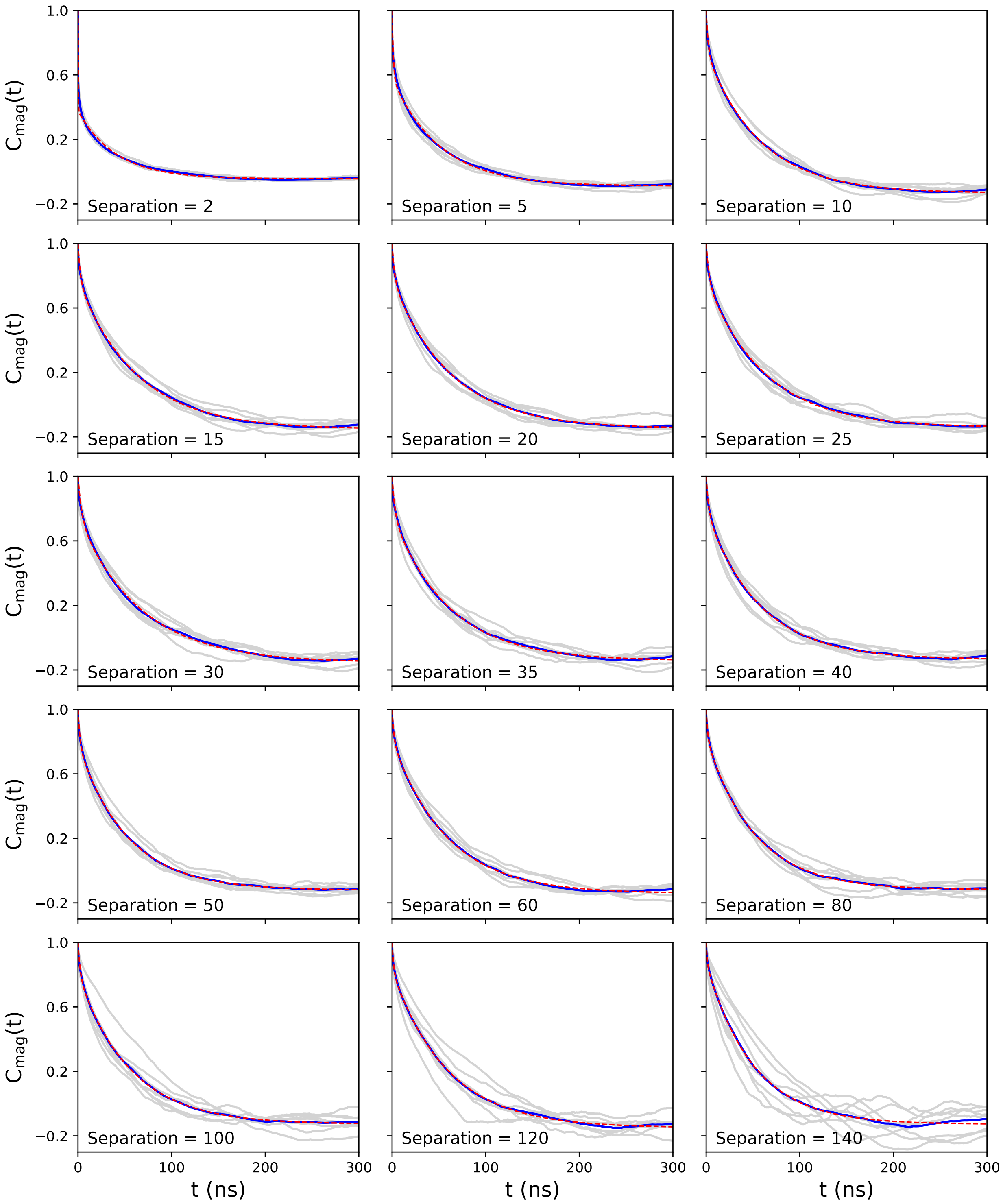

Figure S5: ACFs of the $C_\alpha - C_\alpha$ distance fluctuations at selected sequence separations in the condensate system. The light grey curves represent the 8 individual proteins in the system. The bold blue line is the average ACF over the 8 individual ones. The dashed red line denotes the biexponential fit.

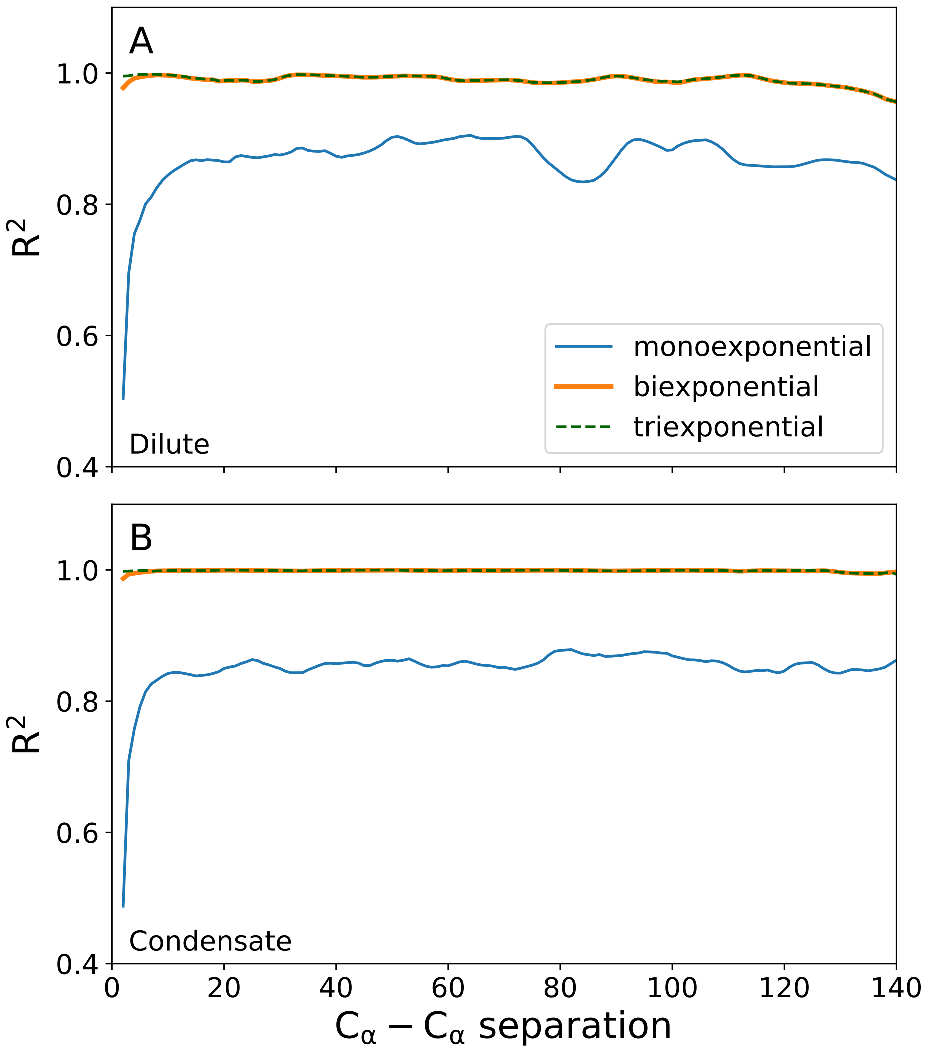

Figure S6: Coefficient of determination (R^2) for mono-, bi-, and triexponential fits of the ACFs of $C_\alpha - C_\alpha$ distance fluctuations as a function of $C_\alpha - C_\alpha$ separation in (A) dilute and (B) condensate systems.

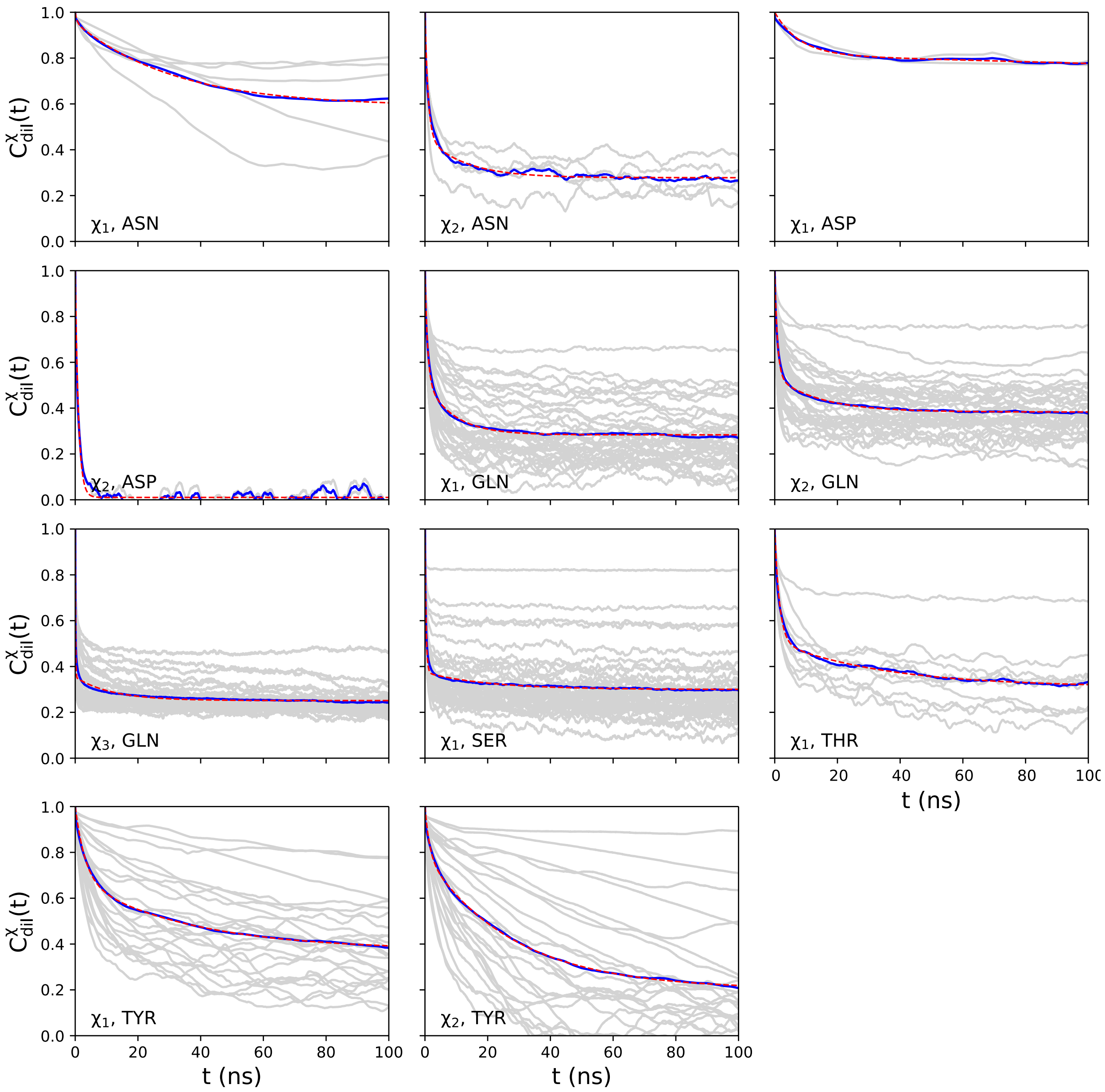

Figure S7: Side-chain dihedral autocorrelation functions of the protein in the dilute system. The light grey curves represent the individual side-chains of the same type present in the system. The bold blue line is the average ACF. The dashed red line denotes the biexponential fit to the mean ACF.

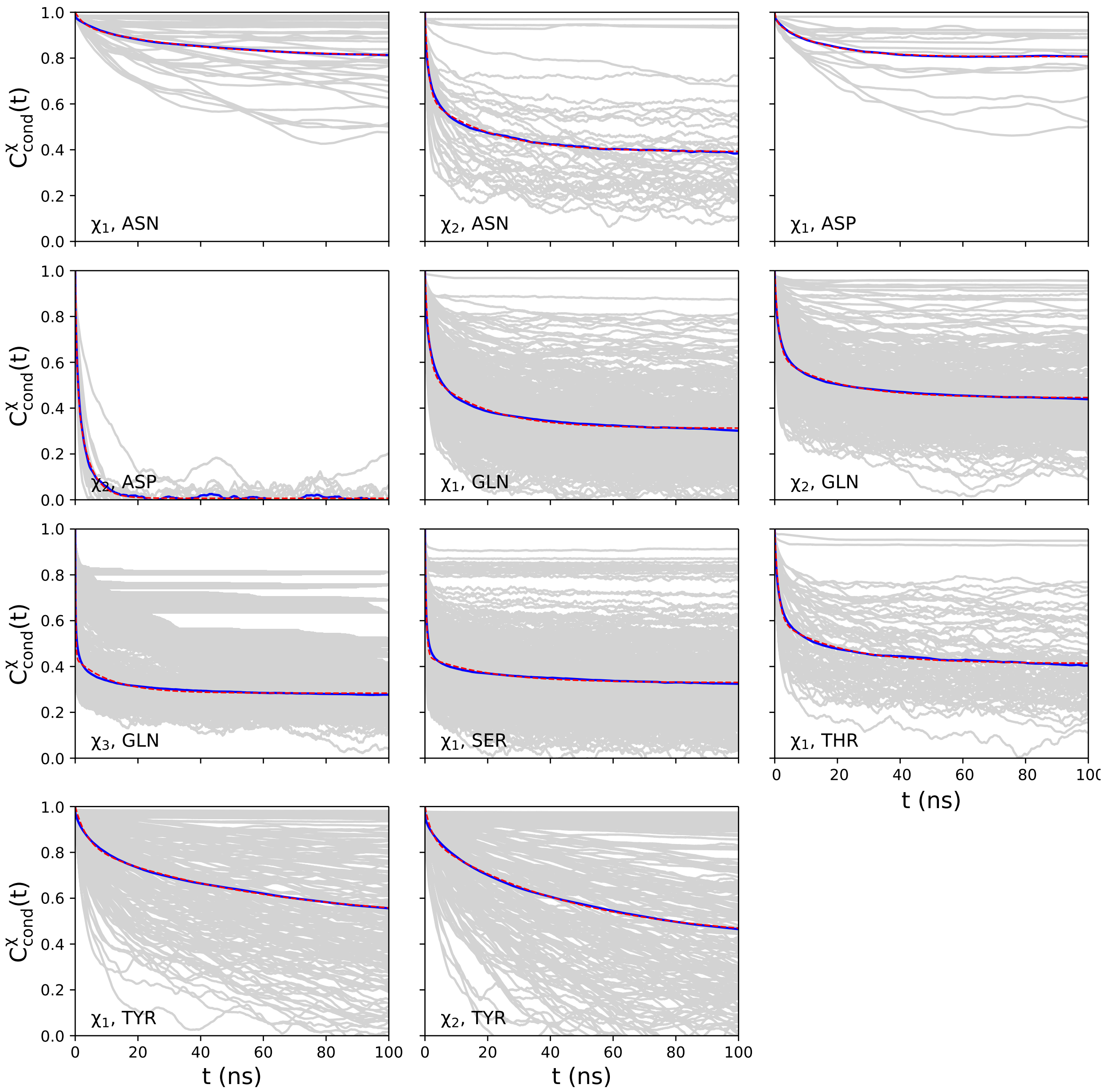

Figure S8: Side-chain dihedral ACFs in the condensate system. The light grey curves represent the individual side-chains of the same type present in the system. The bold blue line is the average ACF. The dashed red line denotes the biexponential fit.

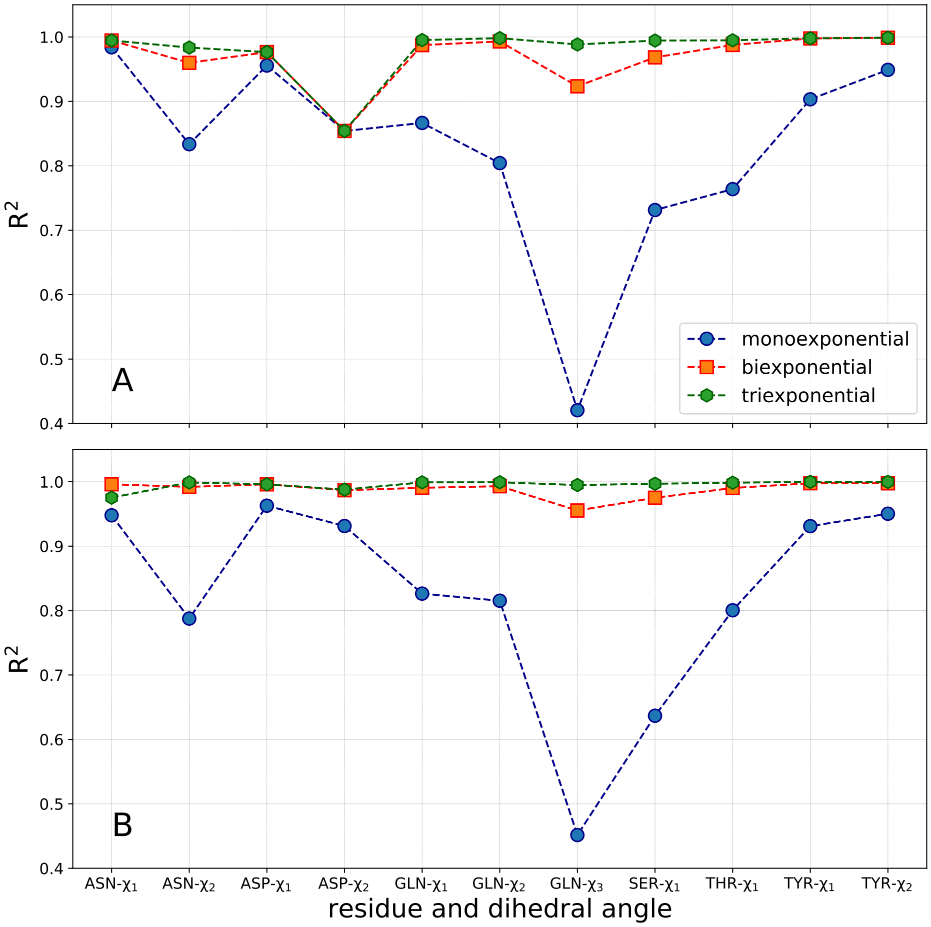

Figure S9: Coefficient of determination (R^2) for mono-, bi-, and triexponential fits of the ACFs of dihedral rotations in the (A) dilute and the (B) condensate systems.

References

(S1) Jumper, J.; Evans, R.; Pritzel, A.; Green, T.; Figurnov, M.; Ronneberger, O.; Tunyasuvunakool, K.; Bates, R.; Žídek, A.; Potapenko, A.; et al. Highly accurate protein structure prediction with AlphaFold. *Nature* **2021**, *596*, 583–589.

(S2) Varadi, M.; Anyango, S.; Deshpande, M.; Nair, S.; Natassia, C.; Yordanova, G.; Yuan, D.; Stroe, O.; Wood, G.; Laydon, A.; et al. AlphaFold Protein Structure Database: Massively expanding the structural coverage of protein-sequence space with high-accuracy models. *Nucleic Acids Res.* **2022**, *50*, D439–D444.

(S3) Mukherjee, S.; Schäfer, L. V. Thermodynamic forces from protein and water govern condensate formation of an intrinsically disordered protein domain. *Nat. Commun.* **2023**, *14*, 5892.

(S4) de Jong, D. H.; Singh, G.; Bennett, W. F. D.; Arnarez, C.; Wassenaar, T. A.; Schäfer, L. V.; Periole, X.; Tieleman, D. P.; Marrink, S. J. Improved parameters for the Martini coarse-grained protein force field. *J. Chem. Theory Comput.* **2013**, *9*, 687–697.

(S5) Benayad, Z.; von Bülow, S.; Stelzl, L. S.; Hummer, G. Simulation of FUS protein condensates with an adapted coarse-grained model. *J. Chem. Theory Comput.* **2021**, *17*, 525–537.

(S6) Berendsen, H. J. C.; Postma, J. P. M.; van Gunsteren, W. F.; DiNola, A.; Haak, J. R. Molecular dynamics with coupling to an external bath. *J. Chem. Phys.* **1984**, *81*, 3684–3690.

(S7) de Jong, D. H.; Baoukina, S.; Ingólfsson, H. I.; Marrink, S. J. Martini straight: Boosting performance using a shorter cutoff and GPUs. *Comput. Phys. Commun.* **2016**, *199*, 1–7.

(S8) Robustelli, P.; Piana, S.; Shaw, D. E. Developing a molecular dynamics force field for both folded and disordered protein states. *Proc. Natl. Acad. Sci. U.S.A.* **2018**, *115*, E4758–E4766.

(S9) Wassenaar, T. A.; Pluhackova, K.; Böckmann, R. A.; Marrink, S. J.; Tieleman, D. P. Going backward: a flexible geometric approach to reverse transformation from coarse grained to atomistic models. *J. Chem. Theory Comput.* **2014**, *10*, 676–690.

(S10) Sarthak, K.; Winogradoff, D.; Ge, Y.; Myong, S.; Aksimentiev, A. Benchmarking Molecular Dynamics Force Fields for All-Atom Simulations of Biological Condensates. *J. Chem. Theory Comput.* **2023**, *12*, 3721–3740.

(S11) Abraham, M. J.; Murtola, T.; Schulz, R.; Páll, S.; Smith, J. C.; Hess, B.; Lindahl, E. GROMACS: High performance molecular simulations through multi-level parallelism from laptops to supercomputers. *SoftwareX* **2015**, *1*, 19–25.

(S12) Bussi, G.; Donadio, D.; Parrinello, M. Canonical sampling through velocity rescaling. *J. Chem. Phys.* **2007**, *126*, 014101.

(S13) Darden, T.; York, D.; Pedersen, L. Particle mesh Ewald: An $N \cdot \log(N)$ method for Ewald sums in large systems. *J. Chem. Phys.* **1993**, *98*, 10089–10092.

(S14) Burke, K. A.; Janke, A. M.; Rhine, C. L.; Fawzi, N. L. Residue-by-residue view of in vitro FUS granules that bind the C-terminal domain of RNA polymerase II. *Mol. Cell* **2015**, *60*, 231–241.

(S15) Murthy, A. C.; Dignon, G. L.; Kan, Y.; Zerze, G. H.; Parekh, S. H.; Mittal, J.; Fawzi, N. L. Molecular interactions underlying liquid- liquid phase separation of the FUS low-complexity domain. *Nat. Struct. Mol. Biol.* **2019**, *26*, 637–648.

(S16) Yeh, I.-C.; Hummer, G. System-Size Dependence of Diffusion Coefficients and Viscosities from Molecular Dynamics Simulations with Periodic Boundary Conditions. *J. Phys. Chem. B* **2004**, *108*, 15873–15879.
