# Supplementary material for: Heterogeneous Slowdown of Dynamics in the Condensate of an Intrinsically Disordered Protein
Source: J Phys Chem Lett. 2024 Nov 1;15(45):11244–51. doi: 10.1021/acs.jpclett.4c02142 (PMC11571228; doi:10.1021/acs.jpclett.4c02142)
Supplement: Supplementary file 2 — jz4c02142_si_002.pdf [file jz4c02142_si_002.pdf]

jz-2024-02142s.R1

Name: Peer Review Information for "Heterogeneous Slowdown of Dynamics in the Condensate of an Intrinsically Disordered Protein"

First Round of Reviewer Comments

Reviewer: 1

Comments to the Author

Major advance reported:

Study by Mukherjee et al. deals with the dynamics of FUS LCD, in solution and condensate, respectively, studied by full atom molecular dynamics simulations. The authors observe that the formation of the condensate suppresses certain modes of motion more than the others. Specifically, the local dynamics of side chains being far less supressed by the condensate than backbone dynamics, especially when greater chain length is considered. Authors suggest this observation can be transferred to other IDP protein condensates.

Immediate significance:

The study is well technically executed and reads very well. I would've gladly recommended it to "publish as is" in a specialized journal.

However, in context of a letter, I'm not convinced it brings significant new physical insights. For example, Zheng et al. 2020 (10.1021/acs.jpcb.0c10489) have already dealt with simulations of protein condensates of this system, using even longer simulations than what the authors present in this manuscript. The 2020 study had focused more on solvent dynamics, however, the issue of solute dynamics had already been considered and extensively discussed by Galvanetto et al. 2023 (10.1038/s41586-023-06329-5). Both studies are cited by the authors, so I assume they are aware of them.

Technical suggestion:

p3 l7: certain --> of certain

Reviewer: 2

Comments to the Author

In the letter "Heterogeneous Slowdown of Dynamics in the Condensate of an Intrinsically Disordered Protein", Mukherjee and Schäfer have used all-atom MD simulations to study the effect of a crowded environment on the dynamics of FUS, an RNA binding protein. There is a nice introduction to biocondensates that is compact, well-written and accessible to the broader audience. Yet it does not lead us to the actual motivation for this study. Overall the authors have not adequately emphasized the importance of their results. In particular, was there anything that was unexpected, or striking? And finally, the authors need to discuss the importance of the results in the context of functionality.

The authors have compared the condensate system, with multiple protein molecules packed together to a dilute system and observed the dynamical retardation of the system. Using various autocorrelation functions, they observed a difference in the retardation of the global and local dynamics.

As argued from Figure 1, the dynamical retardation is more pronounced at longer length scales (i.e taking larger masses) as well as longer lag times. While the Figure has been described in detail, the importance and relevance of the result is not discussed. The same is true for the other figures as well. Why does it matter that the retardation is heterogenous and that side chain motions perhaps do not feel the effect of crowding as much as the overall translational motion of the entire molecule. What is the significance of a 100 fold retardation vs. a 30 fold retardation. Do the numbers themselves have any significance, can they be verified (maybe with some other system that is similar).

In general, finding a direct link between the dynamics and biological function on the basis of an in silico study is always challenging, as asserted by the authors on at least two occasions in this letter. However, I would urge the authors to discuss what is the main impact of this study, and how it can guide experimental groups, and how it actually expands our understanding of IDPs and biocondensates.

Reviewer: 3

Comments to the Author

Summary: The manuscript submitted by Mukherjee and Schäfer investigates the dynamics of an intrinsically disordered human fused in sarcoma (FUS) protein within the condensates at different time and length scales. To this end, they have employed all-atom molecular dynamics simulations using the amber99SB-disp force field at temperature T = 300 K. They find that the translation motion of the protein chains in the condensate, characterized by their mean-squared displacement (MSD), significantly slows down (~2 orders of magnitude) at longer length scales, as compared to their motion in the dilute phase. However, they find that the time scales of side-chain rotamer dynamics, characterized by its auto-correlation function (ACF), remain less affected in the condensates as compared to that in the dilute phase. In line with this, they also find that the timescale derived from the ACF of the dipole moment vector based on individual water molecules slows down two-fold as compared to a six-fold slowdown in the timescale of collective water dynamics computed from the ACF of the total dipole moment. Based on these observations, the authors conclude there is a heterogeneous slowdown in dynamics, with large-scale motions (motions at the chain level) drastically retarded as compared to the small-scale motion (motions at the residue level).

I cannot recommend this article for publication in its current form. However, it may be published provided the authors satisfactorily address my following major comments and revise the manuscript accordingly:

1. I found it difficult to understand the novelty of this work. A recent study (using experiments and computer simulations) that appeared in Nature (https://doi.org/10.1038/s41586-023-06329-5) showed that mesoscopic properties like viscosity and translational diffusion coefficient indicated drastic slowdown within condensates as compared to dilute phase, while the microscopic reconfiguration times remained rapid. Is it not the same conclusions that the authors are deriving here? If so, what new insights does this study provide? Also, in the case of water dynamics, there are recent studies that show their drastic slowdown in condensates as compared to the dilute phase. Please see https://doi.org/10.1021/acs.jpclett.3c02790 and https://doi.org/10.1021/jacs.3c10862. I must note that the authors cite these literature articles as a supporting reference to what they observe. I suggest the authors clearly state in the introduction the scientific gaps that this work tries to address in light of these literature articles.

2. Did the authors try to compute the translational diffusion coefficient for FUS chains in the dense phase for the force field that they use and verify if it matches the experimental diffusivity? As a reference, see https://doi.org/10.1021/acs.jpcb.0c10489.

3. For the orientational ACF of the $CC_{\alpha\alpha}-CC_{\alpha\alpha}$ vectors (Fig. S2), I would have expected that the relaxation time scale corresponds to the time when ACF at least reached $1/ee$, assuming that the decay is purely exponential. If the ACF shows stretched exponential behavior, one could then use a biexponential function like the authors have done. But I see that the ACFs do not decay for larger separations. For example, for a separation of 140, the ACF has not decayed even to 0.9. This is also true in the case of side-chain dihedral $(\chi\chi_1)$ ACF for the residues ASN & ASP (Fig. S6 and S7). Then, how can one reliably get relaxation times for these? What do the reported time scales correspond

to? I believe that the noise in Fig. 2B is because of estimating time scales based on ACFs that have not decayed.

4. In light of my previous comment, I only see error bars reported in Fig. 1. The time scales from the ACFs in the rest of the plots do not show the error estimates. I suggest the authors to estimate

the errors based on individual chains or based on replicates if multiple independent replicas were simulated? I believe this will lead to reliably describing the trends observed in the time scales obtained based on the ACFs.

5. For computing water dynamics within the protein hydration layer, the authors have picked water molecules that are within 0.5 nm distance from the protein surface. How did the authors achieve this for computing ACFs if the water molecules that will be tagged at one time instant can leave the hydration layer in the next few time instants?

A few minor comments:

1. In the abstract, “We found a heterogeneous retardation of the protein dynamics, with large amplitude motions being strongly slowed by up to two orders of magnitude, whereas small-scale motions, such as local backbone fluctuations and side-chain rotations, are less affected.” – Please indicate that the slowdown is in comparison to the dilute phase
2. On page 3, “To fulfil their biological functions, biocondensates need to ensure a high local concentration certain biomolecules” – of certain biomolecules?
3. On page 3, the use of the term “effect of crowding” is confusing as no crowders were used in the study. Please rephrase.
4. Fig 4B could be shown on a semi-log scale.

In general, the manuscript is well-written and can be considered for publication in The Journal of Physical Chemistry Letters after extensive revision in light of the above comments.

Author's Response to Peer Review Comments:

Dear Editor:

We thank you and the Reviewers for the time and efforts spent to evaluate our manuscript and for the positive and constructive feedback, which helped us to further improve our manuscript. In particular, triggered by the comments from the reviewer, we calculated the diffusion coefficient of the FUS chains in the condensate and found it to be within the range of experimental values, which validates our simulation approach. Furthermore, we reworked the text in order to better explain the motivation of our study in light of the current state of the research field, and the relevance of our findings. We hope that in its current revised form, our manuscript can be accepted for publication in JPCL.

Please find the point-by-point replies to the reviewers attached.

Kind regards,

Lars Schäfer

Point-by-point replies

Reviewer 1

Comment: Study by Mukherjee et al. deals with the dynamics of FUS LCD, in solution and condensate, respectively, studied by full atom molecular dynamics simulations. The authors observe that the formation of the condensate suppresses certain modes of motion more than the others. Specifically, the local dynamics of side chains being far less supressed by the condensate than backbone dynamics, especially when greater chain length is considered. Authors suggest this observation can be transferred to other IDP protein condensates.

Immediate significance:

The study is well technically executed and reads very well. I would've gladly recommended it to "publish as is" in a specialized journal.

However, in context of a letter, I'm not convinced it brings significant new physical insights. For example, Zheng et al. 2020 (10.1021/acs.jpcb.0c10489) have already dealt with simulations of protein condensates of this system, using even longer simulations than what the authors present in this manuscript. The 2020 study had focused more on solvent dynamics, however, the issue of solute dynamics had already been considered and extensively discussed by Galvanetto et al. 2023 (10.1038/s41586-023-06329-5). Both studies are cited by the authors, so I assume they are aware of them.

Reply: We thank the Reviewer for directing our attention to this aspect because we realised that in the original manuscript, we might not have explained the main aims of the present study in a sufficiently clear way. Here, we approach biomolecular condensates from a fundamental biophysical chemistry perspective and use FUS-LCD as a model system to obtain new physical insights into which dynamic modes of the (intrinsically disordered) protein are affected how, and by how much, by the dense environment of the condensate. To the best of our knowledge, this question has not yet been thoroughly addressed in the literature, and systematic studies are lacking. Indeed, as pointed out by the reviewer, the Zheng et al. (2020) paper is a very good work and they achieved impressively long simulation times (through the use of the Anton2 supercomputer provided by DE Shaw research, which unfortunately is not available to everybody). However, in addition to focusing also more on solvent dynamics, Zheng et al. did neither set out to identify specific dynamic modes of the protein that are affected by the condensate environment, nor did they characterise the nature of the dynamic protein response to the self-crowding in the droplets. Instead, they provide a detailed and insightful analysis of the ion partitioning between dense and dilute phases, and they also analysed residue-residue contacts that stabilise the droplets, which is not our focus in the present manuscript. Concerning the work of Galvanetto et al (2022), they used fluorescence spectroscopy (single-molecule FRET and nanosecond FCS) in combination with MD simulations to investigate the ProTα–H1 system, which is very different from the FUS-LCD studied in our work. ProTα has ultrahigh affinity towards H1 due to strong electrostatic interactions of the two highly charged proteins (the net charges of ProTα and H1 are -44 and +53, respectively). In strong contrast, the FUS-LCD only has 2 aspartates (net charge -2) and can thus be expected to behave differently. A main finding of Galvanetto et al. was that nanosecond-timescale dynamics are retained in the condensate despite a ca. 300-fold higher viscosity than in the dilute phase, a conclusion that generally agrees with our findings (and also the results reported by Zheng et al, for example). However, also in that work, no specific dynamic protein modes were identified, nor were backbone and sidechain motions separately addressed, as we do in the present study. Finally, we would also like to mention the recent study of Guseva et al. (2023), Ref 49 of the revised manuscript, who performed NMR relaxation experiments of measles virus N_{TAIL}, an IDP that also undergoes LLPS. They showed that while sampling of the protein backbones is not strongly affected in the dense phase, librational, backbone torsional, and segmental (or chainlike) dynamics are considerably slower, which would

remain to be reconciled with the results of Galvanetto et al. Along the same lines, a timeresolved fluorescence spectroscopy study showed that α-synuclein has reduced chain flexibility under LLPS conditions compared to dilute solution (http://doi.org/10.1038/s41557-0200465-9).
Taken together, we thus think that more (and more detailed) studies are desired, and that our present work does indeed provide new physical insights into the nature of the dynamic slowdown of protein dynamics in the FUS-LCD droplets, including the time- and length-scale dependence of the retardation of the identified motional modes, backbone vs. side-chains, etc.
We have added new sections to the text on p. 3 and 4 of the revised manuscript to explain these aspects.

Comment: Technical suggestion: p3 l7: certain → of certain

Reply: Thank you for pointing this out. We have corrected the typo in the revised manuscript.

Reviewer 2

Comment: In the letter “Heterogeneous Slowdown of Dynamics in the Condensate of an Intrinsically Disordered Protein”, Mukherjee and Sch ̈afer have used all-atom MD simulations to study the effect of a crowded environment on the dynamics of FUS, an RNA binding protein. There is a nice introduction to biocondensates that is compact, well-written and accessible to the broader audience. Yet it does not lead us to the actual motivation for this study.

Reply: We agree with the Reviewer and have added an additional section to the Introduction, which also aims at embedding the present work better into the context of the current literature (see p. 3/4 of the revised manuscript). Please see also our reply to Reviewer 1 above.

Comment: Overall the authors have not adequately emphasized the importance of their results. In particular, was there anything that was unexpected, or striking?

Reply: Maybe, at first sight, the finding that side-chain rotamer jump dynamics are not slowed in the condensate was somewhat surprising. However, considering the results obtained from the backbone dynamics analyses, it can be understood (we have added a brief discussion along these lines to the main text on p. 11 of the revised manuscript).
We agree with the reviewer that the way we presented our results in the original manuscript did not optimally emphasize the importance of our findings. We trust that this is now improved by the several revisions done, which were also triggered by the comments by the other Reviewers.

Comment: And finally, the authors need to discuss the importance of the results in the context of functionality.

Reply: The biological function of the N-terminal part of FUS is in transcriptional activation, and of the C-terminal region in protein and RNA binding. So for directly studying biological function one would at least have to explicitly include nucleic acid molecules as well. The one-component ”FUS-only” condensate studied here (and also in many other works) is rather a model system,

which is well-suited to investigate basic biophysical chemistry questions but maybe less for studying biological function. However, as the Reviewer knows, in general protein dynamics are known to be of functional importance in all kinds of contexts, as we discuss in the Introduction and also in the Conclusions part of the manuscript. This was also nicely put by Galvanetto, Schuler, and coworkers in their recent Nature paper: *"The behaviour we observe is an example of the subtle balance of intermolecular interactions in biomolecular phase separation. On the one hand, the interactions must be strong enough for the formation of stable condensates; on the other hand, they need to be sufficiently weak to enable translational diffusion and liquid-like dynamics within the dense phase and molecular exchange across the phase boundary — processes that are essential for function, such as biochemical reactions occurring in condensates."* Along the same lines, our present work is another such example, with a major strong point being that our simulations yield unprecedented microscopically detailed insights into the relationship between the nature of the motions, their amplitudes and time-scales, and the dynamic retardation that the different dynamic modes experience in the dense condensate environment. For example, for FUS functionality, the ability to have rapid side-chain and local backbone fluctuations may be important for the binding of FUS to nucleic acids or transcription factors. We have rewritten the final paragraph of our manuscript to highlight this aspect more clearly.

Comment: The authors have compared the condensate system, with multiple protein molecules packed together to a dilute system and observed the dynamical retardation of the system. Using various autocorrelation functions, they observed a difference in the retardation of the global and local dynamics.

As argued from Figure 1, the dynamical retardation is more pronounced at longer length scales (i.e taking larger masses) as well as longer lag times. While the Figure has been described in detail, the importance and relevance of the result is not discussed. The same is true for the other figures as well. Why does it matter that the retardation is heterogenous and that side chain motions perhaps do not feel the effect of crowding as much as the overall translational motion of the entire molecule. What is the significance of a 100 fold retardation vs. a 30 fold retardation. Do the numbers themselves have any significance, can they be verified (maybe with some other system that is similar).

Reply: We agree with the reviewer that a 100-fold retardation versus a 30-fold retardation might not be that significant. But whether there is a 200-fold (translational diffusion) or a 2-fold slowdown (side chain rotations) might well matter. The focus of our work is not so much on the precise numbers but rather on the basic physicochemical principles, that is, the revealed link between the motional amplitudes and the time-scales of the motions on the one hand, and the (large differences between the) dynamic retardations that the different motions experience in the crowded condensate environment on the other hand.
Concerning the question by the reviewer about whether the numbers can be verified, we followed a suggestion by Reviewer 3 (see below) and calculated the translational diffusion coefficient of the FUS-LCD chains in the condensate from our MD simulations. The value is in agreement with

experimentally measured diffusion coefficients from Fawzi et al. We take this result as support of the accuracy of the computational model, including the design and setup of the simulation system and the force field used.

Comment: In general, finding a direct link between the dynamics and biological function on the basis of an in silico study is always challenging, as asserted by the authors on at least two occasions in this letter. However, I would urge the authors to discuss what is the main impact of this study, and how it can guide experimental groups, and how it actually expands our understanding of IDPs and biocondensates.

Reply: We hope to have addressed this aspect in our answers to the questions above. We have changed the manuscript accordingly, as described.

Reviewer 3

Comment: The manuscript submitted by Mukherjee and Sch ̈afer investigates the dynamics of an intrinsically disordered human fused in sarcoma (FUS) protein within the condensates at different time and length scales. To this end, they have employed all-atom molecular dynamics simulations using the amber99SB-disp force field at temperature T = 300 K. They find that the translation motion of the protein chains in the condensate, characterized by their mean-squared displacement (MSD), significantly slows down (2 orders of magnitude) at longer length scales, as compared to their motion in the dilute phase. However, they find that the time scales of side-chain rotamer dynamics, characterized by its auto-correlation function (ACF), remain less affected in the condensates as compared to that in the dilute phase. In line with this, they also find that the timescale derived from the ACF of the dipole moment vector based on individual water molecules slows down two-fold as compared to a six-fold slowdown in the timescale of collective water dynamics computed from the ACF of the total dipole moment. Based on these observations, the authors conclude there is a heterogeneous slowdown in dynamics, with large-scale motions (motions at the chain level) drastically retarded as compared to the small-scale motion (motions at the residue level). I cannot recommend this article for publication in its current form. However, it may be published provided the authors satisfactorily address my following major comments and revise the manuscript accordingly.

I found it difficult to understand the novelty of this work. A recent study (using experiments and computer simulations) that appeared in Nature (https://doi.org/10.1038/s41586-023-06329-5) showed that mesoscopic properties like viscosity and translational diffusion coefficient indicated

drastic slowdown within condensates as compared to dilute phase, while the microscopic reconfiguration times remained rapid. Is it not the same conclusions that the authors are deriving here? If so, what new insights does this study provide? Also, in the case of water dynamics, there are recent studies that show their drastic slowdown in condensates as compared to the dilute phase. Please see https://doi.org/10.1021/acs.jpclett.3c02790 and https://doi.org/10.1021/jacs.3c10862. I must note that the authors cite these literature articles as a supporting reference to what they observe. I suggest the authors clearly state in the introduction the scientific gaps that this work tries to address in light of these literature articles.

Reply: Concerning our main topic (protein dynamics), we have added a new section to the text that aims at better embedding the motivation and main results of our present work into the context of the literature (see p. 3/4 of the revised manuscript). Please see also our reply to the comment by Reviewer 1 above.

Concerning water dynamics, in the recent JACS paper Lorenz-Ochoa and Baiz (10.1021/jacs.3c10862) used 2D-IR spectroscopy experiments to investigate the picoseconds timesscale dynamics of three vibrational modes of a poly-l-arginine peptide and the nucleic acid adenosine monophosphate (AMP), the arginine sidechain C-N stretch, an AMP ring mode, and the amide backbone carbonyl stretch. For each vibrational mode, which represent local probes, dynamics are slowed down about 2-6 fold between the dilute phase and the condensate phase. Water dynamics were extracted from MD simulations, which show that a fraction of water molecules are highly constrained within the condensate, explaining the observed slowdown in the H-bond dynamics. These findings are in qualitative agreement with our previous work (e.g., https://doi.org/10.1038/s41467-023-41586-y). However, it should be kept in mind that Lorenz-Ochoa and Baiz analysed the water dynamics in terms of the autocorrelation (”survival”) functions of H-bonds *between water and protein groups* (such as C=O and N-H), as these H-bond dynamics primarily drive the 2D-IR relaxation. The other study mentioned by the Reviewer (Krevert et al, 10.1021/acs.jpclett.3c02790) is similar, as they probed the hydrogen-bond dynamics sensed by condensate proteins using 2D-IR spectroscopy of the protein amide I vibrations. The picosecond timescale decays of the frequency correlation of the amide I vibrations were found to be slower in the condensate than in dilute solution, which Krevert et al found to be linked to slowed hydration dynamics in the condensate, which were again probed by MD simulations in that work in terms of protein–water H-bond survival functions. Taken together, we think it is clear that these papers have a different focus from our water analysis (the final part of the results), which exclusively addresses the water molecules in the H-bond network and how this dynamic network is modulated by the (dense) protein environment in the condensate (Figure 4). However, we agree with the Reviewer in that the above-discussed papers should not be cited in the way we did in our original manuscript, and we have changed the text accordingly in the revised manuscript (on p. 12 and p. 14).

Comment: Did the authors try to compute the translational diffusion coefficient for FUS chains in the dense phase for the force field that they use and verify if it matches the experimental diffusivity? As a reference, see https://doi.org/10.1021/acs.jpcb.0c10489.

Reply: We thank the Reviewer for this suggestion. We estimated the diffusion coefficient of the FUS chains from the slope of a linear fit to the MSD curve, which we have added as new Figure S2 to the SI. The obtained diffusion coefficient of $0.26 \cdot 10^{-3}$ nm^2/ns is within the range of experimental values of FUS-LCD condensates reported by Fawzi et al. (0.17 to $0.4 \cdot 10^{-3}$ nm^2/ns, Figure 3 in 10.1021/acs.jpcb.0c10489). We have added a brief description to the main text (on p. 5). However, we consider our calculated diffusion coefficient to be only a rather rough estimate, because 1) the fit assumes a linear relationship between MSD and lag time (time-exponent $\alpha = 1$) and thus neglects subdiffusive behavior, which is expected to play a role for the confined motions in the condensate, 2) it is associated with some statistical uncertainty (see the cyan shaded area in Figure S2), and 3) we did not apply the analytical correction by Hummer et al. to account for finite box size effects because the viscosity of the simulation box is unknown. Such correction would increase the value slightly.

Comment: For the orientational ACF of the $C_\alpha - C_\alpha$ vectors (Fig. S2), I would have expected that the relaxation time scale corresponds to the time when ACF at least reached $1/e$, assuming that the decay is purely exponential. If the ACF shows stretched exponential behavior, one could then use a biexponential function like the authors have done. But I see that the ACFs do not decay for larger separations. For example, for a separation of 140, the ACF has not decayed even to 0.9. This is also true in the case of side-chain dihedral (χ_1) ACF for the residues ASN & ASP (Fig. S6 and S7). Then, how can one reliably get relaxation times for these? What do the reported time scales correspond to? I believe that the noise in Fig. 2B is because of estimating time scales based on ACFs that have not decayed.

Reply: The time scales of some of the dynamic modes are indeed very slow, such that the extracted relaxation times are comparable to the simulation time and need to be estimated from the onset of the decay of the time-correlation functions, as e.g. shown in Figure S3 (previous Figure S2) for the CA-CA reorientation motions and in Figs. S7, S8 (previous Figs. S6, S7) for the side-chain dihedral jumps. We have added error bars to the revised plots in order to show the uncertainties, which are large for some of the slowest correlation times. However, we would like to emphasize that the precise values of the correlation times are not of major importance for our conclusions, but it is merely the observation that certain dynamic modes are much more strongly slowed down compared to others, which is a robust finding.
Concerning the question of what the reported time scales correspond to, we prefer not to link any physical interpretation to the correlation times, e.g., in terms of assigning them to correspond to a particular physical motion (or so). Instead, we consider them to be only fitting parameters. Fig. S3 shows that the fitted simple analytical functions describe the actual data very well.

Comment: In light of my previous comment, I only see error bars reported in Fig. 1. The time scales from the ACFs in the rest of the plots do not show the error estimates. I suggest the authors to estimate the errors based on individual chains or based on replicates if multiple independent

replicas were simulated? I believe this will lead to reliably describing the trends observed in the time scales obtained based on the ACFs.

Reply: We have now added error bars to Figs. 2 and 3. In the former, the error bars represent the standard errors over the timescales derived from the eight proteins in the condensate system (Figs. 2B and 2E). Since the dilute system has only one protein, error bars were not added to Figs. 2A and 2D. In Fig. 3, the error bars denote the standard errors derived over all residues of a certain type and their respective $\chi_{1/2/3}$ angles. We have modified the figure captions to include the description of the errors.

Comment: For computing water dynamics within the protein hydration layer, the authors have picked water molecules that are within 0.5 nm distance from the protein surface. How did the authors achieve this for computing ACFs if the water molecules that will be tagged at one time instant can leave the hydration layer in the next few time instants?

Reply: The protocol for computing the rotational dynamics of hydration water begins with calculation of the residence times of the individual water molecules in the hydration layer, given by the region within 0.5 nm from protein surface. This involved monitoring the entry and exit times of the molecules into/from this PHL. The rotational autocorrelation functions were calculated only for the duration when a water molecule was within in the PHL region. Molecules that transiently visited the hydration layer were omitted from the calculation. We now clarify this on p. 12 of the revised manuscript.

Minor Comments

Comment: In the abstract, “We found a heterogeneous retardation of the protein dynamics, with large amplitude motions being strongly slowed by up to two orders of magnitude, whereas smallscale motions, such as local backbone fluctuations and side-chain rotations, are less affected.” –

Please indicate that the slowdown is in comparison to the dilute phase

Reply: Thanks for pointing this out. We have now modified the sentence accordingly.

Comment: On page 3, “To fulfil their biological functions, biocondensates need to ensure a high local concentration certain biomolecules” – of certain biomolecules?

Reply: We have corrected this typo in the revised manuscript.

Comment: On page 3, the use of the term “effect of crowding” is confusing as no crowders were used in the study. Please rephrase.

Reply: We rephrased the text (at several places) to avoid this possible misunderstanding.

Comment: Fig 4B could be shown on a semi-log scale.

Reply: We now plot the data in Fig. 4B on a semi-log scale.

Comment: In general, the manuscript is well-written and can be considered for publication in The Journal of Physical Chemistry Letters after extensive revision in light of the above comments.

Reply: We thank the Reviewer for the positive evaluation of our work and the constructive feedback.
